# How secondhand smoke exposure affects tobacco use and smoking susceptibility of adolescents: Sex and school differences

**DOI:** 10.18332/tid/140094

**Published:** 2021-09-01

**Authors:** Xiaochen Yang, Zhongheng Yan, Gang Xu, Yinliang Tan, Jingfen Zhu

**Affiliations:** 1 School of Public Health, Shanghai Jiao Tong University School of Medicine, Shanghai, People’s Republic of China; 2 The Ninth People’s Hospital, Shanghai Jiao Tong University School of Medicine, Shanghai, People’s Republic of China

**Keywords:** secondhand smoke, tobacco use, smoking susceptibility, adolescent, gender

## Abstract

**INTRODUCTION:**

Secondhand smoke (SHS) exposure affects tobacco related health behaviors during adolescence and persists into adulthood. This study aimed to investigate the influence of SHS exposure on tobacco use among adolescents stratified by school and gender, and provide recommendations for controlling tobacco use in youth.

**METHODS:**

Through stratified random cluster sampling, 12278 selected students (aged 13–18 years) from schools in China were administered questionnaires. Multiple logistic regression was used to analyze whether SHS exposure would increase the smoking risk and susceptibility of adolescents.

**RESULTS:**

The prevalence of SHS exposure among the participating students was 74.8%. Adolescents exposed to SHS were at higher odds of being susceptible and currently smoking. Students with SHS exposure at both home and public places accounted for 36.6%, greatly increasing the current smoking risk and smoking susceptibility. Home SHS exposure had greater impact on the current tobacco use of boys (OR=2.13; 95% CI: 1.50–3.03) and junior school students (OR=4.67; 95% CI: 2.41–9.06). Exposure from public places increased the risk of current smoking in boys (OR=4.20; 95% CI: 2.31–7.65) and smoking susceptibility of vocational school students (OR=1.51; 95% CI: 1.07–2.15). Students with highlevel exposure to SHS had 2.25 times higher odds of e-cigarette use.

**CONCLUSIONS:**

The prevalence of SHS exposure is still high among adolescents in China and is associated with increased risk for tobacco use regardless of gender and school level. Effective smoke-free strategies should be developed and strictly implemented. Boys and junior school students constitute vulnerable populations exposed to SHS at home.

## INTRODUCTION

Tobacco use is currently one of the most serious public health threats worldwide. Tobacco use and related behaviors, including secondhand smoke (SHS) exposure, smoking susceptibility, and exposure to tobacco advertising, harm health and impose a heavy economic burden on society^1-3^. Smoking susceptibility precedes smoking behavior and is defined as not taking a firm decision against cigarette smoking^4^. It acts as a predictor of smoking experimentation and smoking status, with adolescents less likely to respond to tobacco prevention programs^5^. Prior research has indicated that smoking susceptibility is used as a significant independent predictor of future initiation into tobacco use^6^.

Despite the approval of the World Health Organization (WHO) Framework Convention on Tobacco Control (FCTC) in 2005, China is now the world’s leading nation in both tobacco production and consumption^7^, with more than 300 million smokers and 740 million non-smokers exposed to SHS^8^. Over the past few decades, China has been carrying out tobacco prevention interventions including cigarette sale age restrictions, media campaigns, and school health education courses that target middle school and high school students^9^. The current smoking prevalence among adolescents in junior high schools was 3.9% in 2019 and the rates for senior and vocational high school students were 8.6% and 14.7%, respectively^10^. The prevalence of smoking among junior high school students in 2019 was lower than that in 2014 (6.9%)^10^, which might prove tobacco control efforts effective, but this rate was still high.

The smoking behavior of adolescents is affected by personal, psychological, and social factors such as gender, smoking behavior of peers or family members and social environment^11,12^. SHS exposure is one of the important risk factors that might increase susceptibility to smoking and nicotine dependence among those who have begun smoking^13^. Prior studies have indicated that SHS could influence tobacco use and smoking behaviors^14-16^. Review studies found that SHS exposure was associated with increased susceptibility and initiation of smoking^17^. According to one of the nicotine dependence theories, SHS exposure could result in repeated nicotine exposure and tolerance to nicotine’s adverse effects, thereby contributing to nicotine-induced behavioral changes^18^.

Research has shown that an adolescent who smokes a first cigarette is more likely to continue into adulthood and have greater difficulty quitting^19^. Considering the harmful effects of smoking in adolescents, how SHS exposure induces adolescents to initiate smoking warrants further clarification. In a high-density urban setting, the passive exposure to SHS in adolescents can be much higher than the smoking prevalence of the general population^20^. According to a study conducted in the United States, homes are the settings where the highest percentage of non-smokers have reported daily SHS exposure^12^. Compared with adults, private settings are probably major and neglected sources of exposure for children and adolescents. Therefore, identifying the characteristics of SHS exposure among adolescents is of great importance. The conflicting reports on the disparities of gender and school in adolescent smoking behavior support the need for more research with regard to the relationship between SHS exposure and tobacco use^11,21,22^. How specific levels of sources of exposure affect young people of different genders also needs more research, since more boys than girls become more susceptible to initiating smoking as they age^23^.

Less is known about how the levels or sources of SHS exposure affect e-cigarette use, which currently presents a high prevalence among adolescents. Prior studies have proven that SHS exposure at home mediates family smoking and e-cigarette use of adolescents^24^. The correlations between SHS exposure and e-cigarette use might present diversified stratification by gender and school.

A number of studies have examined the factors associated with tobacco use and initiation of smoking among adolescents. But the specific relationship between SHS exposure and tobacco related behaviors among those aged 13–18 years is scant and should be examined. With the high prevalence of SHS exposure and harmful role it plays in tobacco related behaviors in adolescents, the current study aimed to investigate the relationship between SHS exposure and tobacco use behaviors stratified by sex and school among a representative sample of adolescents in Shanghai, China. This study should assist in identifying adolescents who are at high risk of smoking initiation and inform interventions to improve targeted enforcement of smoke-free policies.

## METHODS

### Study population and data collection

This study was a cross-sectional study with two-stage stratified cluster sampling to select representative samples of school students aged 13–18 years in Shanghai from September 2017 to January 2018. In the first stage, the sixteen selected districts were stratified according to urban and rural areas, and four districts were randomly selected. Huangpu and Putuo were selected as central urban areas, and Minhang and Jiading as non-central urban areas. In the second stage, all schools in these four districts were further stratified based on school type and 33 schools were randomly selected. A total of 12422 students from 33 schools in 4 districts were invited to participate in this study, and 12278 (98.8%) valid questionnaires were included in the analysis.

The self-administered questionnaire was adapted from the WHO Global Youth Tobacco Survey. The online questionnaires were administered during regular class sessions and students were asked to complete them anonymously and independently. Trained research members briefed students on the details of the items in the questionnaire. The other details regarding the panel have been published^25^. This study was approved by the Ethics Committee of the Shanghai Jiao Tong University (SJUPN-201703; approved on December 5, 2017).

### Measures

We defined participants as current smokers if they reported smoking in the last 30 days. Ever smokers were defined as those who had ever tried a cigarette but not in the past 30 days. The susceptibility to smoking in the future among never smokers was assessed by two questions: ‘Do you think you will smoke a cigarette in the next year?’ and ‘If your best friend offers you a cigarette, may you smoke it?’. Participants who answered ‘definitely not’ to both questions were classified as non-susceptible to smoking while the others were classified as susceptible. Use of electronic cigarettes were measured using the question: ‘Have you tried e-cigarettes (even one puff)?’; with those who responded ‘yes’ defined as ever users.

Exposure to SHS at home, in indoor public places, and at outdoor areas was measured by five-point ordinal scale: ‘0 days’, ‘1–2 days’, ‘3–4 days’, ‘5–6 days’ and ‘all 7 days’ during the past seven days. Those who reported ‘0 days’ to all three places were identified as not having SHS exposure. Respondents who were exposed to SHS in public places were referred to those reporting more than 0 days exposure to indoor or outdoor places. To investigate the effects of different exposure levels, the total score of these three questions was used as a continuous variable and divided equally into low, medium, and high SHS exposure levels, respectively. To create distinct groups based on the source of SHS exposure, adolescents were categorized into the following four groups: 1) no SHS exposure, 2) only exposure at home, 3) only exposure in public places, and 4) SHS exposure in both places.

Sociodemographic covariates included age, gender, school type, residence status, household registration (local, non-local), pocket money, academic performance and smoking status of close friends and parents.

### Statistical analysis

All statistical analyses were performed using IBM SPSS 22.0 and the complex sample analysis was used. A weighting factor was applied to each student record to adjust for non-response and for varying probabilities of selection including selection probability of districts, the number of schools in each district, and the number of students in each school. SHS exposure and tobacco use conditions were presented as percentages. Chi-squared analysis was used to test the relationship between gender and school type both at home as well as in public places. Multiple logistic regression was used to explore the association between SHS exposure and current smoking status, smoking susceptibility as well as e-cigarette use among adolescents. Sex, age, school type, district, boarding, local, GPA, pocket money, friends’ smoking and parents’ smoking were included as covariates. The trend test was used to analyze the relationship between SHS exposure level and tobacco use behaviors. Odds ratios (ORs) and 95% confidence intervals (CIs) are reported. Differences were considered to be statistically significant for p<0.05.

## RESULTS

Among the respondents, 51.6% were males and 48.4% were females. The proportion of junior high school students, senior high school students and vocational school students was 61.99%, 23.67% and 14.34%, respectively, and this was similar to the composition of students in Shanghai. Current smokers and e-cigarette users were 2.52% and 7.67% of the investigated students while smoking susceptibility among nonsmokers was 7.67% (Table 1).

**Table 1:** Basic characteristics of survey subjects (N=12278)

	*Weighted*		*Unweighted*
	*Mean (95% CI)*	*Number*	*Mean (95% CI)*
**Age** (years)	14.28 (14.23–14.31)	670050	14.62 (14.58–14.66)
	***% (95% CI)***	***Number***	***n (%)***
**Type of school**			
Junior high school	61.99 (61.13–62.85)	415377	6462 (52.63)
Senior high school	23.67 (22.88–24.48)	158593	2475 (20.16)
Vocational high school	14.34 (13.86–14.83)	96080	3341 (27.21)
**District**			
Urban	33.36 (33.13–33.60)	223534	4042 (32.92)
Suburban	66.64 (66.40–66.87)	446516	8236 (67.08)
**Gender**			
Male	51.6 (50.69–52.52)	345778	6419 (52.28)
Female	48.4 (47.48–49.31)	324272	5859 (47.72)
**Residence**			
Local	72.21 (71.39–73.02)	483864	8755 (71.31)
Non-local	27.79 (26.98–28.61)	186186	3523 (28.69)
**Boarding in school**			
Yes	13.55 (13.00–14.12)	90791	2302 (18.75)
No	86.45 (85.88–87.00)	579260	9976 (81.25)
**Monthly allowance** (RMB)			
<200	61.23 (60.35–62.11)	173882	7011 (57.10)
200–600	25.95 (25.17–26.75)	85865	3401 (27.70)
>600	12.81 (12.24–13.41)	670050	1866 (15.20)
**GPA**			
Top 25%	32.96 (32.11–33.83)	220860	3924 (31.96)
Average	46.28 (45.38–47.20)	310129	5712 (46.52)
Bottom 25%	20.75 (20.03–21.5)	139061	2642 (21.52)
**Parents’ smoking**			
None	36.56 (35.68–37.44)	244962	4377 (35.65)
One	59.25 (58.35–60.14)	396985	7364 (59.98)
Both	4.19 (3.85–4.57)	28103	537 (4.37)
**Friends’ smoking**			
None	82.79 (82.13–83.43)	554740	9693 (78.95)
Some	15.16 (14.55–15.79)	101550	2274 (18.52)
Most or all	2.05 (1.83–2.31)	13760	311 (2.53)
**Smoking status**			
Never	92.12 (91.64–92.57)	617244	11091 (90.33)
Ever	5.36 (4.98–5.76)	35893	780 (6.35)
Current	2.52 (2.28–2.80)	16913	407 (3.31)
**Smoking susceptibility**			
No	92.33 (91.86–92.78)	618651	11140 (90.73)
Yes	7.67 (7.22–8.14)	51399	1138 (9.27)
**E-cigarette use**			
No	92.33 (91.86–92.78)	618651	11524 (93.86)
Yes	7.67 (7.22–8.14)	51399	754 (6.14)

CI: confidence interval. GPA: grade point average. RMB: 100 Chinese Renminbi about US$15.

The prevalence of reported SHS exposure among adolescents in our sample was 74.8%, and that at home and public places was 41.5% and 69.9%, respectively. The rate of SHS exposure in indoor public places was 59.3% and at outdoor public places was 63.5%. More male students reported being exposed to high levels of SHS (30.4%) compared to females (26.9%, p<0.001). Vocational students with SHS exposure both at home and public places accounted for 43.4%, which was significantly higher than junior and senior high school students (34.7% and 37.4%, p<0.001). The prevalence of overall SHS exposure among adolescents from different schools showed significant differences (χ^2^=7.97, p<0.05), and similarly that of home (χ^2^= 58.56, p<0.001) and public places (χ^2^=13.62, p<0.01) (Table 2).

**Table 2:** Secondhand smoke exposure rate among adolescents stratified by sex and school (N=12278)

	*Sex*				*High school*					*Total*
	*Male*	*Female*	*χ2*	*p*	*Junior*	*Senior*	*Vocational*	*χ2*	*p*	
	*% (95% CI)*	*% (95% CI)*			*% (95% CI)*	*% (95% CI)*	*% (95% CI)*			*% (95% CI)*
**Total SHS**										
No	26.0 (24.9–27.1)	24.4 (23.3–25.6)	4.08	0.050	24.9 (23.9–26.0)	24.4 (22.7–26.1)	27.9 (26.4–29.4)	7.97	0.014	25.2 (24.4–26.0)
Yes	74.0 (72.9–75.1)	75.6 (74.4–76.7)			75.1 (74.0–76.1)	75.6 (73.9–77.3)	72.1 (70.6–73.6)			74.8 (74.0–75.6)
**SHS from home**										
No	57.7 (56.4–58.9)	59.3 (58.0–60.6)	3.47	0.071	60.5 (59.4–61.7)	57.9 (56.0–59.8)	50.6 (48.9–52.3)	58.56	0.000	58.5 (57.6–59.4)
Yes	42.3 (41.1–43.6)	40.7 (39.4–42.0)			39.5 (38.3–40.6)	42.1 (40.2–44.0)	49.4 (47.7–51.1)			41.5 (40.6–42.4)
**SHS from public places**										
No	31.0 (29.9–32.2)	29.2 (28.0–30.4)	4.96	0.031	29.7 (28.6–30.8)	29.1 (27.3–30.9)	33.8 (32.2–35.4)	13.62	0.001	30.1 (29.3–31.0)
Yes	69.0 (67.8–70.1)	70.8 (69.6–72.0)			70.3 (69.2–71.4)	70.9 (69.1–72.7)	66.2 (64.6–67.8)			69.9 (69.0–70.7)
**SHS from indoor public places**										
No	41.1 (39.8–42.3)	40.2 (38.9–41.5)	0.998	0.333	40.7 (39.5–41.9)	40 (38.1–41.9)	41.6 (40.0–43.3)	1.177	0.501	40.7 (39.8–41.6)
Yes	58.9 (57.7–60.2)	59.8 (58.5–61.1)			59.3 (58.1–60.5)	60 (58.1–61.9)	58.4 (56.7–60.0)			59.3 (58.4–60.2)
**SHS from outdoor public places**										
No	37.6 (36.4–38.8)	35.4 (34.1–36.6)	6.626	0.013	37.1 (36–38.3)	33.5 (31.7–35.4)	38.7 (37–40.3)	15.945	<0.001	36.5 (35.6–37.4)
Yes	62.4 (61.2–63.6)	64.6 (63.4–65.9)			62.9 (61.7–64)	66.5 (64.6–68.3)	61.3 (59.7–63)			63.5 (62.6–64.4)
**SHS level**										
Low	36.4 (35.2–37.6)	36.2 (34.9–37.4)	25.89	0.000	36.9 (35.7–38.1)	34.7 (32.9–36.6)	36.4 (34.8–38.0)	12.37	0.009	36.3 (35.4–37.2)
Medium	33.2 (32.0–34.4)	37.0 (35.7–38.3)			35.3 (34.1–36.4)	35.8 (34.0–37.7)	32.6 (31.0–34.2)			35.0 (34.2–35.9)
High	30.4 (29.2–31.6)	26.9 (25.7–28.0)			27.8 (26.8–28.9)	29.5 (27.7–31.3)	31.0 (29.5–32.6)			28.7 (27.9–29.5)
**SHS home and public**										
No	26.0 (24.9–27.1)	24.4 (23.3–25.6)	14.80	0.003	24.9 (23.9–26.0)	24.4 (22.7–26.1)	27.9 (26.4–29.4)	111.78	0.000	25.2 (24.4–26)
Home only	5.0 (4.5–5.6)	26.9 (25.7–28.0)			4.7 (4.2–5.3)	4.7 (4–5.6)	6.0 (5.2–6.8)			4.9 (4.5–5.3)
Public places only	31.7 (30.5–32.9)	4.8 (4.2–5.4)			35.6 (34.4–36.8)	33.5 (31.7–35.4)	22.7 (21.4–24.2)			33.3 (32.4–34.1)
Both	37.3 (36.1–38.5)	35.9 (34.6–37.1)			34.7 (33.6–35.9)	37.4 (35.5–39.3)	43.4 (41.8–45.1)			36.6 (35.7–37.5)

The associations between SHS exposure and the risk of current smoking and smoking susceptibility of adolescents are presented in Tables 3 and 4. Overall, the risk of adolescents’ tobacco use behaviors was greater the higher the exposure level. Male students with high-level SHS exposure had higher current smoking risk (OR=3.53; 95% CI: 2.26–5.50) and smoking susceptibility (OR=1.66; 95% CI: 1.18– 2.23) compared with female students. The current smoking risk was the highest in senior high school students with high-level SHS exposure (OR=4.01; 95% CI: 1.56–10.30) than other students. Vocational high school students were most susceptible to smoking (OR=1.83; 95% CI: 1.22–2.75). Analysis of continuous exposure to SHS showed that the risk of current smoking was increased for any gender and school with more SHS exposure. Exposure to SHS in only one place, whether home or public place, showed no significant relationship with tobacco use risk, while SHS exposure at both places greatly increased the students’ current smoking risk and smoking susceptibility. Stratified by sex and school, home SHS exposure had a greater impact on the current smoking behavior and smoking susceptibility of boys (OR=2.13; 95% CI: 1.50–3.03) and junior high school students (OR=4.67; 95% CI: 2.41–9.06). SHS exposure in public places increased the current smoking risk in boys (OR=4.20; 95% CI: 2.31–7.65) and the smoking susceptibility of vocational school students (OR=1.51; 95% CI: 1.07–2.15).

**Table 3:** ORs for current smoking by secondhand smoke exposure stratified by sex and school (N=12278)^a^

	*Now smoking*	*Total*	*Sex*		*High school*		
	*% (95% CI)*		*Male*	*Female*	*Junior*	*Senior*	*Vocational*
**SHS home**							
No (Ref.)	1.2 (1–1.4)	1	1	1	1	1	1
Yes	4.4 (3.9–5.0)	2.45 (1.80–3.33)	2.13 (1.50–3.03)	1.60 (1.06–2.41)	4.67 (2.41–9.06)	1.34 (0.71–2.53)	2.56 (1.74–3.76)
**SHS public**							
No (Ref.)	1.2 (0.9–1.6)	1	1	1	1	1	1
Yes	3.1 (2.8–3.5)	1.34 (0.93–1.93)	4.20 (2.31–7.65)	0.77 (0.37–1.60)	0.80 (0.39–1.64)	2.23 (0.93–5.34)	1.28 (0.85–1.94)
**SHS level**							
Low (Ref.)	1.1 (0.8–1.4)	1	1	1	1	1	1
Medium	1.3 (1.0–1.6)	1.04 (0.70–1.56)	1.31 (0.83–2.06)	0.56 (0.23–1.33)	0.64 (0.23–1.79)	1.07 (0.38–2.99)	1.30 (0.84–2.01)
High	5.9 (5.2–6.7)	3.26 (2.23–4.76)	3.53 (2.26–5.50)	2.70 (1.31–5.57)	3.79 (1.64–8.76)	4.01 (1.56–10.3)	2.65 (1.73–4.04)
p for trend		<0.001	<0.001	0.013	0.001	0.003	<0.001
SHS (cont.)		1.16 (1.12–1.20)	1.15 (1.10–1.19)	1.19 (1.11–1.27)	1.23 (1.13–1.33)	1.21 (1.12–1.30)	1.09 (1.05–1.13)
**SHS home and public**							
No (Ref.)	1.3 (1.0–1.7)	1	1	1	1	1	1
Home only	0.8 (0.4–1.7)	0.51 (0.20–1.31)	0.54 (0.19–1.57)	0.49 (0.04–5.76)	0.48 (0.06–3.88)	N/A	1.10 (0.42–2.88)
Public only	1.1 (0.8–1.4)	0.82 (0.52–1.30)	1.03 (0.61–1.74)	0.40 (0.16–1.03)	0.28 (0.09–0.86)	1.52 (0.54–4.24)	0.91 (0.54–1.55)
Both	4.9 (4.3–5.5)	2.63 (1.71–4.05)	2.77 (1.68–4.57)	2.59 (1.15–5.82)	2.51 (1.02–6.17)	2.50 (0.89–7.05)	2.85 (1.78–4.58)

OR: odds ratio.

a Model adjusted for age, sex, school type, district, boarding, local, GPA, pocket money, friends’ smoking and parents’ smoking.

**Table 4:** ORs for future smoking intention by secondhand smoke exposure stratified by sex and school among never smokers (N=11091)^a^

	*Smoking susceptibility*	*Total*	*Sex*		*High school*		
	*% (95% CI)*		*Male*	*Female*	*Junior*	*Senior*	*Vocational*
**SHS home**							
No (Ref.)	3.1 (2.7–3.6)	1	1	1	1	1	1
Yes	5.1 (4.5–5.8)	1.46 (1.14–1.87)	1.73 (1.25–2.40)	1.13 (0.76–1.67)	2.00 (1.32–3.03)	0.95 (0.61–1.48)	1.28 (0.90–1.83)
**SHS public**							
No (Ref.)	3.5 (3.0–4.2)	1	1	1	1	1	1
Yes	4.1 (3.7–4.6)	0.97 (0.76–1.23)	0.93 (0.68–1.28)	0.99 (0.69–1.43)	0.95 (0.61–1.48)	0.94 (0.61–1.44)	1.51 (1.07–2.15)
**SHS level**							
Low (Ref.)	3.0 (2.5–3.6)	1	1	1	1	1	1
Medium	3.7 (3.1–4.3)	1.18 (0.92–1.52)	1.31 (0.94–1.83)	1.02 (0.70–1.50)	1.42 (0.95–2.13)	0.86 (0.55–1.36)	1.31 (0.89–1.93)
High	5.5 (4.7–6.3)	1.51 (1.16–1.97)	1.66 (1.18–2.33)	1.27 (0.82–1.97)	1.51 (0.97–2.34)	1.28 (0.79–2.07)	1.83 (1.22–2.75)
p for trend		0.001	0.002	0.190	0.026	0.243	0.009
SHS (cont.)		1.04 (1.01–1.08)	1.06 (1.02–1.10)	1.02 (0.97–1.07)	1.04 (1.00–1.09)	1.04 (0.98–1.11)	1.05 (1.00–1.09)
**SHS home and public**							
No (Ref.)	3.3 (2.7–4.0)	1	1	1	1	1	1
Home only	4.7 (3.2–6.9)	1.43 (0.86–2.36)	1.78 (0.96–3.31)	0.93 (0.38–2.31)	2.90 (1.50–5.64)	0.55 (0.16–1.82)	0.65 (0.27–1.53)
Public only	3.0 (2.5–3.6)	0.97 (0.73–1.29)	0.96 (0.65–1.41)	0.94 (0.62–1.43)	0.97 (0.61–1.54)	0.83 (0.51–1.36)	1.26 (0.81–1.97)
Both	5.2 (4.5–5.9)	1.43 (1.07–1.90)	1.64 (1.13–2.38)	1.12 (0.71–1.78)	1.69 (1.03–2.77)	0.87 (0.53–1.45)	1.90 (1.23–2.95)

OR: odds ratio.

a Model adjusted for age, sex, school type, district, boarding, local, GPA, pocket money, friends’ smoking and parents’ smoking.

Additionally, the relationship between SHS exposure and e-cigarette use risk was analyzed (Table 5). The odds of e-cigarette use were 2.25 (95% CI: 1.77–2.87) times higher in those who had high exposure to SHS. Female students with home SHS exposure (OR=1.82; 95% CI: 1.18–2.79) and high SHS exposure (OR=3.05; 95% CI: 1.90–4.87) had higher e-cigarette use risk than males. Senior high school students with SHS exposure at public places (OR=2.32; 95% CI: 1.26–4.29) and high SHS exposure (OR=4.32; 95% CI: 2.41–7.74) had higher e-cigarette use risk. Those reporting SHS exposure from both places had 1.89 (95% CI: 1.44–2.47) times increased odds of using e-cigarettes.

**Table 5:** ORs for e-cigarette use by secondhand smoke exposure stratified by sex and school (N=12278)^b^

	*E-cigarette use*	*Total*	*Sex*		*High school*		
	*% (95% CI)*		*Male*	*Female*	*Junior*	*Senior*	*Vocational*
**SHS home**							
No (Ref.)	3.2 (2.8–3.6)	1	1	1	1	1	1
Yes	7.5 (6.8–8.2)	1.53 (1.24–1.89)	1.42 (1.11–1.81)	1.82 (1.18–2.79)	1.27 (0.88–1.84)	1.67 (1.07–2.6)	1.81 (1.36–2.42)
**SHS public**							
No (Ref.)	2.9 (2.4–3.5)	1	1	1	1	1	1
Yes	5.9 (5.4–6.4)	1.31 (1.04–1.64)	1.29 (0.99–1.69)	1.36 (0.89–2.07)	1.36 (0.93–2.01)	2.32 (1.26–4.29)	0.92 (0.68–1.23)
**SHS level**							
Low (Ref.)	2.6 (2.2–3.1)	1	1	1	1	1	1
Medium	3.8 (3.3–4.4)	1.28 (1.00–1.64)	1.20 (0.90–1.61)	1.54 (0.95–2.47)	1.43 (0.93–2.21)	1.56 (0.84–2.90)	1.08 (0.79–1.47)
High	9.4 (8.5–10.4)	2.25 (1.77–2.87)	1.99 (1.50–2.64)	3.05 (1.90–4.87)	2.33 (1.52–3.56)	4.32 (2.41–7.74)	1.45 (1.07–1.96)
p for trend		<0.001	<0.001	<0.001	<0.001	<0.001	0.019
SHS (cont.)		1.10 (1.07–1.12)	1.08 (1.05–1.12)	1.12 (1.07–1.18)	1.09 (1.05–1.14)	1.18 (1.12–1.25)	1.03 (1.00–1.07)
**SHS home and public**							
No (Ref.)	2.9 (2.4–3.5)	1	1	1	1	1	1
Home only	2.9 (1.9–4.2)	0.94 (0.58–1.52)	0.80 (0.45–1.44)	1.34 (0.56–3.20)	0.43 (0.13–1.47)	0.47 (0.06–3.89)	1.65 (0.94–2.88)
Public only	3.4 (2.9–4.0)	1.13 (0.85–1.49)	1.09 (0.78–1.52)	1.23 (0.72–2.10)	1.09 (0.70–1.71)	1.83 (0.92–3.64)	0.89 (0.61–1.30)
Both	8.1 (7.4–8.9)	1.89 (1.44–2.47)	1.72 (1.25–2.36)	2.36 (1.37–4.06)	1.58 (0.99–2.54)	3.36 (1.70–6.65)	1.66 (1.19–2.32)

OR: odds ratio.

b : Model adjusted for age, sex, school type, district, boarding, local, GPA, pocket money, friends’ smoking, parents’ smoking and smoking status.

## DISCUSSION

Our study showed a still high prevalence of SHS exposure among adolescents in Shanghai, China, and the positive association of SHS exposure, especially dual exposure at home and public places, with tobacco use behaviors. We also found the gender and school type disparities in SHS exposure and smoking behaviors and the extent to which these disparities are pronounced over exposure level/source. Our findings might supplement the evidence on the impacts of SHS exposure in adolescents, providing reference for the formulation of targeted prevention and control strategies.

The exposure prevalence of SHS at home and indoor public places among adolescents in Shanghai (41.5% and 59.3%) are apparently lower than those obtained from China’s 2019 national survey (63.2% and 72.0%)^10^. Young populations in our study reported the highest exposure rate at outdoor areas (63.5%), but this rate is also lower than the national level (67.3%). These relatively low exposure rates could be explained by local policies. The Regulations of Shanghai Municipality on smoking control in public places came into effect 1 March 2017 and banned smoking in all indoor areas. Therefore, parents might have better awareness and knowledge in avoiding smoking in families, especially in the presence of their children^26^. However, the indoor clean air laws lacked effective supervision and smoking control at outdoor public places, which remained the main sources of exposure to SHS among adolescents in Shanghai. The SHS exposure rate of adolescents in China is significantly higher than those in developed countries (e.g. home exposure rate 21.7% in the US)^13^.

Exposure to SHS is an independent risk factor of being susceptible to smoking in adolescents. This study indicates that non-smokers who were exposed to SHS at home and in public places had a higher prevalence of susceptibility to smoking than those who were not and the rate increased by level of SHS exposure. These findings are consistent with prior studies^27^.

The separate and combined prevalence of SHS exposure from different sources were studied. It is worth noting that SHS exposure rate from only one source, either home or public place, is relatively low but the exposure rate from multiple sources is surprisingly high. Our study also shows that SHS exposure at one only place had little impact, but exposure at two places significantly increased smoking-related behaviors. This indicates that adolescents are more likely to be exposed to SHS both at home and at public places. Exposed to SHS in multiple places may greatly increase risk of tobacco use among youth.

This study shows SHS exposure source disparities in their impacts on smoking behaviors. Adolescents exposed to household SHS have a higher current smoking risk than those exposed at public places. In terms of smoking susceptibility, exposure to SHS at home also has a greater impact on non-smokers compared with public places. This suggests that SHS exposure at home is more likely to have both immediate and long-term effects on youth, initiating non-smokers into smoking onset, in line with the previous research^28^. Prior studies have shown that adolescents with parental smoking are more likely to be exposed to home SHS^29^. Parental smoking was associated with a higher risk of initiating adolescent offspring into smoking^30^ and SHS exposure may explain the relationship between parental smoking and the beginning of smoking^27^. Considering these conditions, special smoke-free home interventions and strengthening parents’ family anti-smoking awareness may prove effective in restricting adolescents from becoming active smokers from SHS exposure^31-33^.

Our results on impacts of exposure to SHS from different sources on adolescent smoking behaviors show differences in gender and school. Boys exposed to SHS at home or at public places were more significantly affected in terms of current smoking use than girls who conversely had a higher prevalence of SHS exposure in public places. This conclusion is similar to that of the existing studies^22, 28^. It might be due to the social perception that smoking among males is more acceptable than among females. Boys emulating the behaviors of adult males with higher smoking rate, are more likely to initiate smoking^34^. Although SHS exposure was not significantly related to smoking susceptibility in girls, females who were exposed to domestic and high-level SHS had higher odds of smoking currently. The focus of tobacco control in females had changed from SHS in the 1990s to both smoking and SHS in 2017^35^. Studies have suggested that there has been a steady increase in smoking prevalence among young females in China, from 0.29% in 1984 to 18.1% in 2018^35, 36^. Thus, further research targeting factors that influence girl’s smoking behaviors and practical strategies focusing on preventing female adolescents from initiating smoking, are urgently needed.

The risk of current smoking and smoking susceptibility among junior high school students was more significantly affected by SHS exposure at home. According to the theory of human development, family members have a stronger influence on adolescents aged 13–15 years than on those aged 16–17 years^37^. Junior high school students who are younger are likely to imitate the smoking behaviors of intimate people, especially parents. SHS exposure in public places has a greater impact on future susceptibility to smoking for vocational school students, which might be due to the fact that they have more contact with society than other school students, and are thus more susceptible to deleterious environmental influences.

E-cigarette use among youth has gained popularity worldwide^38^ and the prevalence of e-cigarette use among middle school students in China is raising (1.2% in 2014 to 2.7% in 2019)^39^. Identifying youth who are susceptible to e-cigarette use might help in prevention efforts. Current studies reveal that e-cigarette secondhand aerosol (SHA) exposure was associated with susceptibility to e-cigarette use^40^. Our study adds to this that conventional combustible tobacco smoke exposure is also associated with e-cigarette use among almost all groups of adolescents. Females and senior high school students have greater odds of using e-cigarettes.

Considering the strong effects observed of SHS exposure on adolescents’ smoking behaviors, it is necessary to better control the exposure of susceptible adolescents to SHS. Especially, strategic interventions for non-smokers exposed to SHS who are at high risk of smoking susceptibility and prohibiting the transition from susceptibility to smoking behavior, may be an effective smoking prevention measure. As stated by the WHO, only eliminating smoking in indoor spaces fully can protect non-smokers from exposure to SHS. Given the high rate of SHS exposure at outdoor areas, there is concern that the smoking bans should be considered more comprehensively, covering not only indoor workplaces and public places. Apart from general tobacco control legislations, policies should emphasize the protection of adolescents’ health, and impose stringent tobacco control regulations in schools and other places where the minors gather.

### Strengths and limitations

The cross-sectional study design is a limitation, as a causal link between study variables and current smoking status and susceptibility to smoking cannot be established. How factors during adolescence assist in predicting smoking in later adolescence needs clarification using a longitudinal design. Future studies are warranted to compile longitudinal surveillance data to examine smoking susceptibility and initiation. Our study might have been biased, as some possible potential confounding factors were not controlled for. However, the representativeness, large sample size and high response rate among students, enables generalization of the results to the schoolgoing adolescent population in China.

## CONCLUSIONS

Exposure to SHS has significant impact on tobacco use in the younger population, irrespective of gender and type of school. Considering the health and social consequences associated with SHS exposure, tailored public health policies and measures such as health promotion activities and strong anti-smoking regulations are needed to increase awareness of the adverse health effects of smoking, especially among adolescent populations. Society, schools and families should jointly work to create a smoke-free environment, and develop and implement policies for preventing cigarette use among adolescents.

## Data Availability

The data supporting this research are available from the authors on reasonable request.
